# Periampullary Clot Causing Biliary Obstruction: A Rare Presentation

**DOI:** 10.7759/cureus.25490

**Published:** 2022-05-30

**Authors:** Khalid Al Shamousi, Zakariya AL-Naamani, Umaima Al Salmi, Farooq Rehman, Mujahid Al-Busaidi

**Affiliations:** 1 Medicine, Sultan Qaboos University, Muscat, OMN; 2 Medicine, Sultan Qaboos University Hospital, Muscat, OMN

**Keywords:** ampulla, cholestatic jaundice, severe sepsis, acute cholangitis, jaundice cholestatic, endoscopic retrograde cholangiopancreatography (ercp)

## Abstract

Acute cholangitis is an emergency condition that requires an emergency biliary drainage for source control of the infection. Commonly cholangitis is precipitated by biliary obstruction due to causes like stones, strictures, stents, or malignancy of the pancreaticobiliary or ampullary origin. We report a unique case of a man who had acute cholangitis due to a periampullary clot who was fully recovered after clot removal by endoscopic retrograde cholangiopancreatography (ERCP).

## Introduction

Acute cholangitis is a medical emergency with many causes of which biliary obstruction secondary to choledocholithiasis, neoplasms, benign or malignant strictures, and sclerosing cholangitis are some of the common etiologies [1]. Hemobilia is a rare cause of biliary obstruction [2] and classically presents with upper abdominal pain, jaundice, and upper gastrointestinal (GI) bleeding, formally known as Quincke's triad, and only happens altogether in 22%-35% of the cases [3,4].

Causes of hemobilia include iatrogenic, traumatogenic, neoplastic, inflammatory, infectious, and vascular etiologies. Hemobilia is mainly reported as case series since it’s uncommon. Sandblom, Yoshida et al., and Green et al. reported 355, 103, and 222 case series, respectively [3,5,6]. Clot formation in the biliary system can result in complete or partial blockage of the duct; this was described by Sandblom et al. [7]. At times, these clots may become stones [8].

If the bile flow is not interrupted and the clot is in continuous contact with the bite, the clot usually dissolves within 24 hours due to the fibrinolytic properties of the bile [9]. In profuse hemobilia, the blood is usually mixed with the bile, and thus the clot is a mixture of blood and bile and hence less likely to form an organized clot. On the other hand, if the hemorrhage is small and intermittent, then the clot is usually made of pure blood, resulting in organized clot formation leading to biliary obstruction [10].

In the presence of evidence of biliary obstruction and high degree suspicion of hemobilia, it is recommended to proceed with decompression of the biliary system by performing endoscopic sphincterotomy, percutaneous drainage, or use of fibrinolytic agents [11]. Bleeding into the biliary system is usually arterial in origin and less venous (usually happens in the setting of portal hypertension) [12]. Acute cholangitis, pancreatitis, or cholecystitis secondary to hemobilia is described in the literature as case reports [13,14].

## Case presentation

A 31-year-old man was admitted to the hospital with an acute presentation with jaundice for 10 days and altered mental status in the past 24 h. Physical examination revealed patient is deeply jaundiced, gasping, unresponsive on Glass coma scale (GCS) 3/15, tachypneic with a respiratory rate of 40 per minute, desaturated down to 47% at room air, tachycardic with a heart rate of 140 bpm, blood pressure of 74/35. The patient was immediately intubated and resuscitated with Intravenous fluid, started on vasopressor, and antibiotics were given.

**Figure 1:**
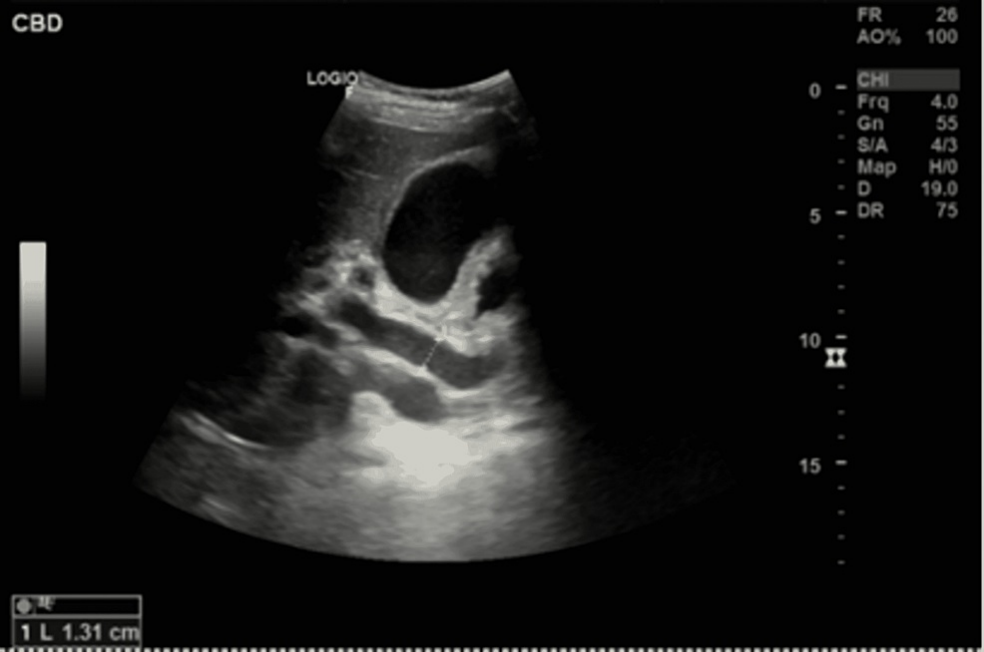
Abdominal ultrasonography showing dilated common bile duct 13m

Laboratory tests revealed disseminated intravascular coagulopathy with an international normalization ratio (INR) of 2.68 (0.90-1.1), activate partial thromboplastin time (aPTT) 88.6 seconds (25-36.4s), prothrombin time (PT) 27.9 (9.8-12.0s), severe thrombocytopenia platelet count of 10 ( 150- 450), leukocytosis of 30.2 (2.2 x 10^9^/L) mainly neutrophils of 26.6, normal hemoglobin. C-reactive protein 245 mg/L (0-5 mg/L), acute renal impairment with creatinine 662 µmol/L (59-140 µmol/L), urea 32.4 mmol/L (2.8-8.1 mmol/L), bicarbonate of 9 mmol/L ( 22-29 mmol/L). His liver function tests (LFTs) showed alanine aminotransferase 57 U/L (0-41), aspartate aminotransferase (71 U/L) (0-40), alkaline phosphatase 923 U/L (40-129 U/L), total bilirubin 419 µmol/L ( 0-17), lipase 1,665 U/L ( 13-60). Acute viral hepatitis A, B, C, and E were all negative. A brain computed tomography (CT) demonstrated an intracranial bleed. Abdominal ultrasound demonstrated common bile duct (CBD) measuring 13 mm with right and left hepatic ducts dilated, gallbladder distended, and showed mild wall edema and no gallbladder stone. Edematous pancreases are suggestive of acute pancreatitis (Figure 1). Unfortunately, because of the acute kidney injury, no cross-sectional images were done to evaluate the pancreaticobiliary system.

**Figure 2:**
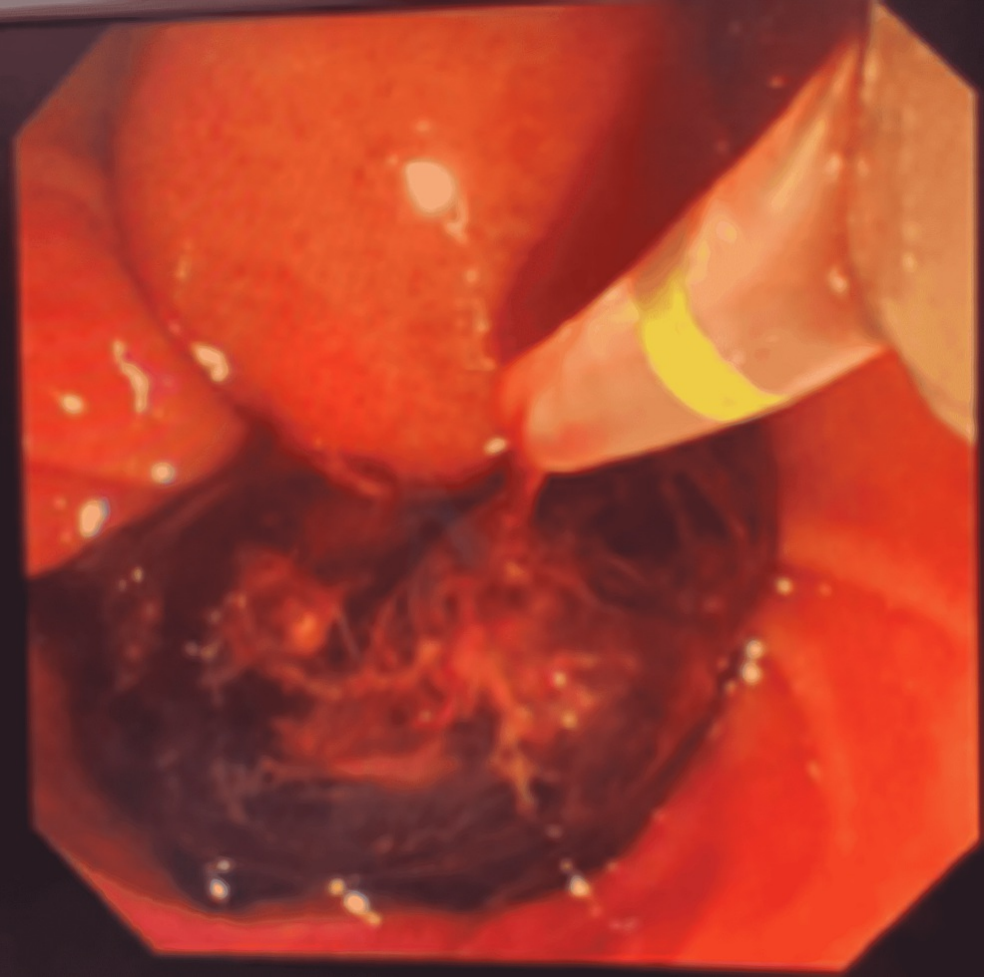
Initial endoscopic image during the ERCP showing a pulged ampulla and the periampullary clot

**Figure 3:**
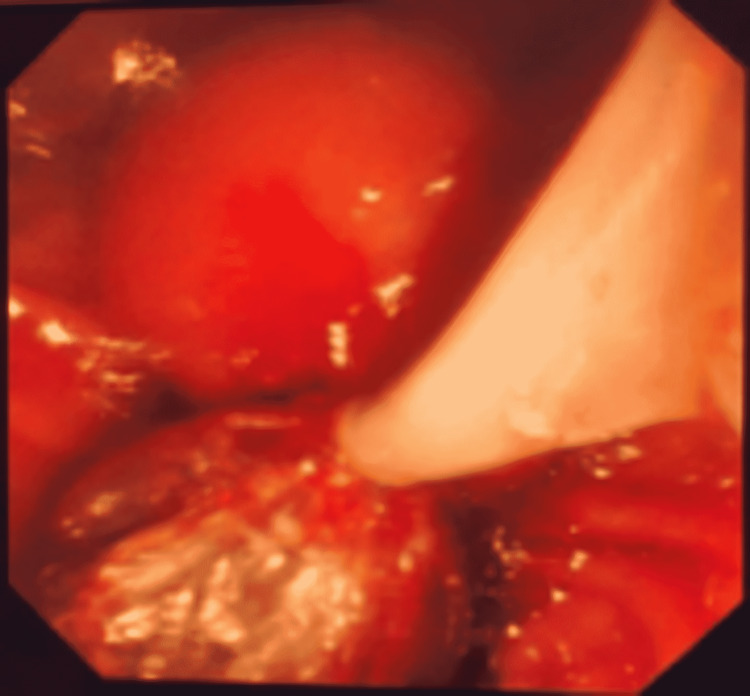
Mobilizing the periampullary clot with a Roth-net

**Figure 4:**
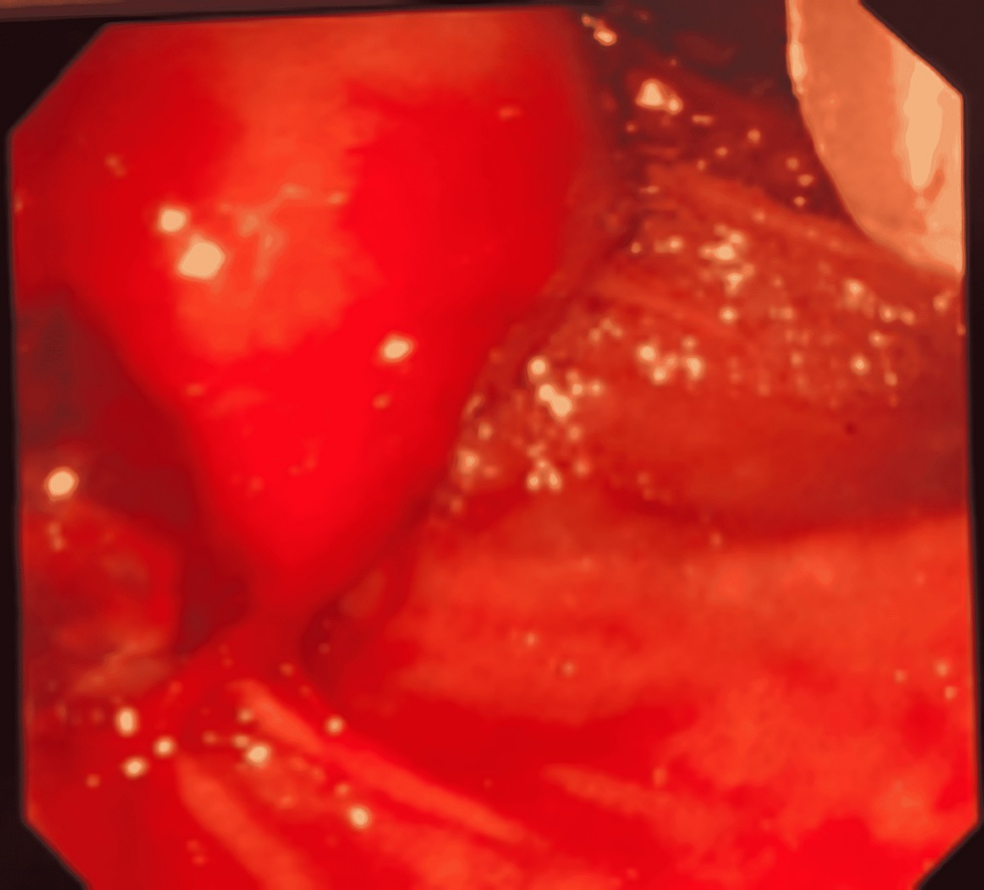
Hemopilia after mobilizing the periampullary clot

Due to sepsis-like presentation, cholangitis with severe sequelae was suspected; thus, an emergency endoscopic retrograde cholangiopancreatography (ERCP) was done. The ampulla appeared plugged with a large clot located at the biliary orifice (Figure 2). Due to severe coagulopathy despite fresh frozen plasma (FFP) administration, thus a pre-cut was avoided. Upon mobilizing the clot with a Roth-net (Figure 3), there was active haemobilia (Figure 4). The visualization was impaired, and the procedure was terminated without biliary cannulation.

**Figure 5:**
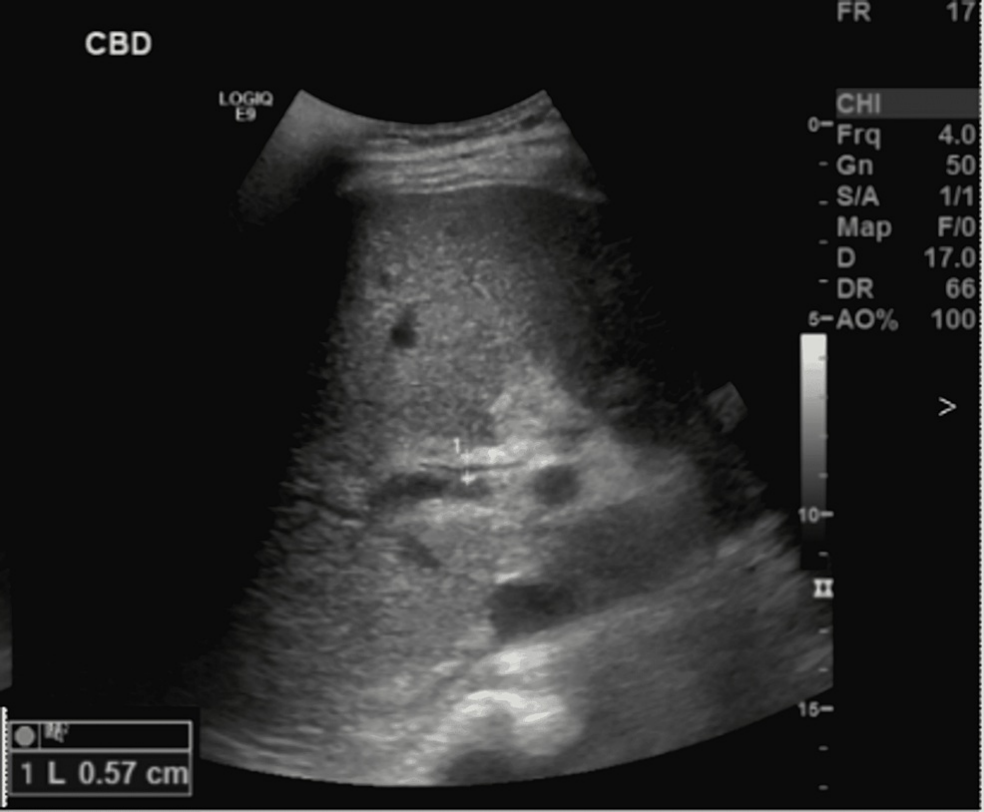
Post-ERCP ultrasound of the abdomen: CBD 5.7 mm

Interestingly, the hepatic cholestatic values did improve (Table 1). An ultrasound abdomen follow-up demonstrated significant improvement of CBD diameter 5.7 mm (was 13 mm on admission) (Figure 5). Moreover, there was a partial improvement in the hemodynamics with decrements in the pressors requirement.

**Table 1:** Trends post-ERCP of liver enzymes, bilirubin, and white blood count ERCP - endoscopic retrograde cholangiopancreatography

	Day 0	Day 1	Day 3	Day 5	Day 14
Alanine transaminase (ALT)	58	58	45	24	12
Aspartate aminotransferase (AST)	103	102	68	24	13
Alkaline phosphatase (ALP)	468	182	110	123	81
Total bilirubin	332	327	208	91	33
White blood count	30.2	16.0	8.6	12.0	8.1

This is the first case of such presentation, a periampullary clot causing severe hepatic obstruction complicated by multi-organ dysfunction. Still, unresolved whether the clot was a prime cause or an aggravator of a cascade that started due to a different etiology. In conclusion, we believe that mobilizing the periampullary clot in this patient led to his improvement. However, the sustained damages to other organs, namely the brain and kidneys, will take time to evolve or resolve.

## Discussion

In our case, a fresh clot was found at the ampulla, and the patient presented with features of acute pancreatitis/cholangitis associated with multi-organ dysfunction and disseminated intravascular coagulopathy (DIC) with very low platelets and high INR. Images showed dilated biliary system and cholestatic picture of liver function tests; hence we proceeded with ERCP. The clot was dislodged mechanically with a Roth-net, but cannulation of the bile duct was unsuccessful due to poor visibility of the ampulla because of bleeding. No sphincterotomy was done. The patient was ventilated, kept on inotropic support, and covered with antibiotics. Blood and platelet transfusion was also given. The next day there was a significant improvement in his liver function tests with a significant drop in bilirubin and alkaline phosphatase and subsequently, there was complete normalization of the LFTs and gradual improvement in his PLT count and coagulopathy as well. The patient was off inotropic support the very next day and was eventually extubated. Repeat ultrasound showed dramatic regression of the biliary dilatation. There was no evidence of ongoing upper GI bleed (hemobilia) later on.

In the literature, we could not find any similar case that improved dramatically after the dislodgment of an ampullary clot. However, it could be expected that some cases may resolve without an endoscopic procedure because such fresh clots may dislodge spontaneously.

It is not clear whether the biliary obstruction was a sequel or a cause of the ampullary clot that we found and removed. However, there was a dramatic improvement in the liver biochemistry and overall clinical as well as the radiological picture after the removal of the clot. Therefore, that clot may have an attributable effect on the clinical and biochemical picture. Further workup was not possible as the patient left against medical advice (LAMA).

## Conclusions

Hemobilia is an uncommon but important cause of upper GI bleeding. Biliary obstruction due to clot formation secondary to hemobilia is rare but should be considered if no other etiology is apparent and there is a history of associated upper GI bleed in the same presentation. Imaging and endoscopy/ERCP are the preferred methods of diagnosis and management. Early recognition and appropriate intervention are crucial.
